# Diagnostic et prise en charge des cystites à éosinophiles

**DOI:** 10.11604/pamj.2018.31.45.16149

**Published:** 2018-09-20

**Authors:** Kays Chaker, Abdelrazek Bouzouita, Marwa Gharbi, Ahlem Blel, Marouene Chakroun, Haroun Ayed, Mohamed Cherif, Mohamed Riadh Ben Slama, Soumaya Rammeh, Amine Derouiche, Mohamed Chebil

**Affiliations:** 1 Service d'Urologie, Hôpital Charles Nicole, Tunis, Tunisie; 2 Service d'Anatomie Pathologique, Hôpital Charles Nicole, Tunis, Tunisie

**Keywords:** Cystite à éosinophile, inflammation, forme pseudo-tumoral, Eosinophilic cystitis, inflammation, pseudotumoral form

## Abstract

La cystite à éosinophiles est une pathologie inflammatoire de la paroi vésicale. Elle est rare, il n'existe pas des recommandations établies concernant sa prise en charge. Il s'agit d'une étude rétrospective ayant concerné dix observations de cystites à éosinophiles diagnostiquées et prises en charge dans notre service entre 2006 et 2017. L'âge moyen des patients était de 46 ans. On a noté une prédominance masculine. Un terrain atopique était noté dans 3 cas. Le mode de présentation le plus fréquent était des signes urinaires irritatifs dans 9 cas, une hématurie macroscopique dans 8 cas et des algies pelviennes dans 6 cas. Une hyper-éosinophilie sanguine était présente dans 4 cas. La cystoscopie avait montré des pétéchies dans 5 cas, un aspect pseudo-tumoral dans 4 cas et était normal dans un cas. Pour les formes pseudo-tumorales une résection endoscopique a été pratiquée. Quatre patients ont été traités par les anti-inflammatoires non stéroïdien, avec amélioration des symptômes. Six malades ont été surveillés. Après un recul moyen de 50 mois, aucune récidive n'a été rapportée. La cystite à éosinophiles est une pathologie rare. La présentation clinique est non spécifique. La prise en charge repose sur des moyens médicaux non invasifs dans les formes peu symptomatiques.

## Abstract (en)

Eosinophilic cystitis is an inflammatory disease of the bladder wall. It is rare, so there are no standard treatment guidelines. We conducted a retrospective study of 10 patients with eosinophilic cystitis diagnosed and treated in our Department between 2006 and 2017. The average age of patients was 46 years; there was a male predominance. Three patients were atopic. The most common symptoms were irritative urinary symptoms in 9 cases, macroscopic haematuria in 8 cases and pelvic pain in 6 cases. Four patients had high levels of eosinophils in their blood. Cystoscopy showed petechiae in 5 cases, pseudotumor in 4 cases. It was normal in one case. Patients with pseudotumor underwent endoscopic resection. Four patients were treated with nonsteroidal anti-inflammatory drugs, with improvement in symptoms. Six patients were monitored. After a mean follow-up interval of 50 months, no recidivism was reported. Eosinophilic cystitis is rare with non-specific clinical manifestations. Patients with little symptomatic eosinophilic cystitis undergo non-invasive medical treatments.

## Introduction

La cystite éosinophile est une maladie de la vessie rare décrite pour la première fois par Edwin Brown en 1960 [1]. Les observations histologiques comprennent une inflammation transmurale de la vessie, principalement avec des éosinophiles. Au stade chronique, on peut également observer une fibrose et une nécrose musculaire pouvant entraîner une contraction de la vessie [2]. La cause et la pathogenèse de la maladie ne sont pas claires et ne sont pas entièrement comprises. Il est spéculé qu'un complexe antigène-anticorps est formé sur l'exposition de l'antigène dans la vessie. Cette réponse induite par les Ig E conduit à la dégranulation des mastocytes, attirant ainsi les éosinophiles et provoquant une réponse inflammatoire avec des lésions tissulaires [3, 4]. De nombreuses étiologies et associations à d'autres maladies ont été proposées [4]. Celles-ci incluent différents médicaments, lésions vésicales, irritation vésicale chronique à la suite d'une chirurgie vésicale, d'une parasitose, d'une allergie aux aliments et aux médicaments, d'une infection des voies urinaires, d'un carcinome urothélial, de troubles auto-immuns et d'une entérite à éosinophiles [4, 5]. Il est important de se concentrer sur cette maladie rare, car la présentation symptomatique et clinique variable des cystites à éosinophiles peut entraîner un retard du diagnostic et du traitement. À son tour, un traitement retardé ou insuffisant des cystites à éosinophiles peut entraîner une gêne accrue pour le patient en raison de la chronicité potentielle de la maladie, ainsi que de la récurrence des symptômes.

## Méthodes

Nous avons réalisé une étude mono centrique rétrospective descriptive, regroupant l'ensemble de patients présentant une cystite à éosinophiles pris en charge à notre institution entre janvier 2006 et décembre 2017. Pour être inclus, les patients devaient être âgés d'au moins 14 ans au moment du diagnostic. Les patients perdus de vue, injoignables par le téléphone ou dont les dossiers sont non exploitables ont été exclus de cette étude. Compte tenu du caractère rétrospectif de l'étude, le type d'examens complémentaires réalisés n'était guidé par aucune recommandation. L'ensemble des données a été colligé à partir des dossiers cliniques à l'aide d'une fiche de recueil standardisée. Celle-ci incluait des données portant sur les données de l'anamnèse, l'examen clinique, examens biologiques courants: numération formule sanguine (NFS), vitesse de sédimentation, CRP, créatininémie. On a aussi recueilli les données endoscopiques, histologiques, thérapeutiques ainsi que évolutives des patients étudiés. Les données cliniques et para cliniques ont été saisies dans un tableau Excel (Microsoft Excel^®^ 2008). Les statistiques descriptives (moyennes, médianes, écart-types), utilisées pour les variables continues et pour le pourcentage des variables qualitatives, ont été déterminées en utilisant les fonctions de calcul de Excel^®^ 2008. En raison du nombre relativement faible de patients, il n'a pas été réalisé d'analyse statistique uni ou multivariée.

## Résultats

**Figure 1:**
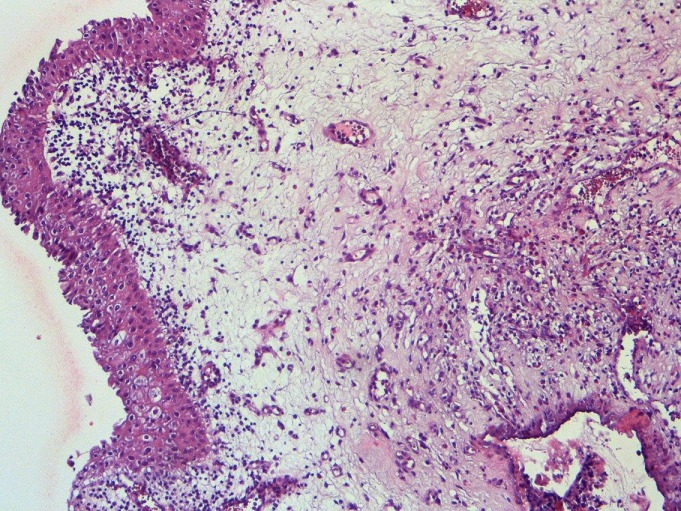
muqueuse vésicale oedémateuse et infiltrée par un dense infiltrat inflammatoire (HE x 100)

**Figure 2:**
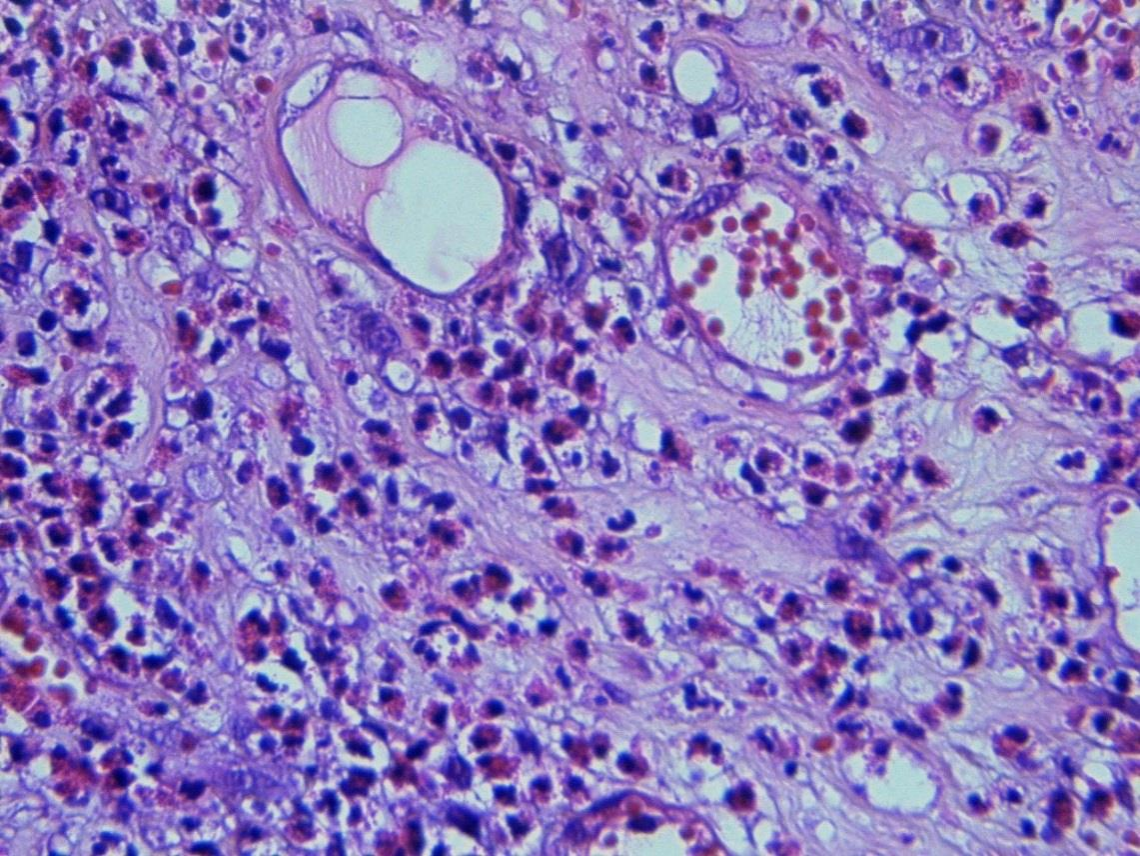
infiltrat inflammatoire constitué de polynucléaires éosinophiles (HE x 400)

L'âge moyen des patients était de 46 ans avec des extrêmes allant de 25 à 68 ans. Le sexe ratio était 3/2. Un terrain atopique était noté dans 3 cas (30%). Le mode de présentation le plus fréquent était des signes urinaires irritatifs dans 9 cas (90%), une hématurie macroscopique dans 8 cas (80%) et des algies pelviennes dans 6 cas (60%). Les urines étaient stériles dans 100% des cas. Une hyper-éosinophilie sanguine était présente dans 4 cas (40%). La cystoscopie avait montré des pétéchies dans 5 cas (50%), un aspect pseudo-tumoral dans 4 cas (40%) et était normal dans un cas (10%). Après résection endoscopique à visée biopsique, l'histologie avait montré infiltration des couches de la paroi vésicale par des éléments inflammatoires au sein desquels prédominent les cellules éosinophiles (Figure 1, Figure 2). Pour les formes pseudo-tumorales une résection endoscopique a été pratiquée. Quatre patients ont été traités par les anti-inflammatoires non stéroïdien, avec amélioration des symptômes. Six malades ont été surveillés. Après un recul moyen de 50 mois, aucune récidive n'a été rapportée. Le Tableau 1 récapitule l'ensemble des patients inclus dans cette étude.

**Tableau 1:** tableau récapitulatif des observations

	Age	Sexe	Terrain atopique	Signes révélateurs	Cystoscopie	Histologie	Taux des éosinophiles	Traitement	Évolution
Observation 1	42 ans	Homme	Asthme	Hématurie Pollakiurie Douleur pelvienne	Formation papillaire	Infiltration de la paroi vésicale par des éléments inflammatoires au sein desquels prédominent les cellules éosinophiles	700/mm^3^	Résection endoscopique	Aucune récidive
Observation 2	38 ans	Femme	-	Hématurie Douleur pelvienne Pollakiurie	Lésions pétéchiales		237/mm^3^	AINS	
Observation 3	68 ans	Homme	Asthme	Hématurie Douleur pelvienne Brulure mictionnelle	Formation papillaire		1120/mm^3^	Résection endoscopique	
Observation 4	57 ans	Homme	-	Brulure mictionnelle Douleur pelvienne	Lésions pétéchiales		80/mm^3^	Pas de Traitement spécifique	
Observation 5	29 ans	Femme	Allergie aux pollens	Hématurie Pollakiurie	Normal		850/mm^3^	AINS	
Observation 6	25 ans	Femme	-	Hématurie Brulure mictionnelle	Lésions pétéchiales		135/mm^3^	AINS	
Observation 7	39 ans	Homme	-	Hématurie Brulure mictionnelle	Lésions pétéchiales		350/mm^3^	AINS	
Observation 8	67 ans	Homme	-	Brulure mictionnelle Pollakiurie Douleur pelvienne	Formation papillaire		54/mm^3^	Résection endoscopique	
Observation 9	45 ans	Femme	-	Hématurie Pollakiurie Brulure mictionnelle	Lésions pétéchiales		925/mm^3^	Pas de Traitement spécifique	
Observation 10	52 ans	Homme	-	Hématurie Douleur pelvienne	Formation papillaire		475/mm^3^	Résection endoscopique	

## Discussion

La cystite à éosinophiles (CE) est une affection rare et exceptionnelle, respectivement, chez les adultes et les enfants. La CE peut survenir à tout âge de 5 jours à 87 ans [6]. Une répartition égale existait entre les hommes et les femmes, mais chez les enfants, les garçons étaient plus souvent touchés que les filles [6]. L'âge médian au moment du diagnostic dans la population pédiatrique est de 6,5 ans, [7] avec un ratio hommes-femmes de 3:1 [3, 6]. Dans la majorité des cas, elle survient sur un terrain immuno-allergique [8]. Une réaction immunologique antigène-anticorps responsable de l'attraction d'éosinophiles dans la paroi vésicale qui libèrent des cytokines responsables du processus inflammatoire [4]. Une uropathie préexistante ou des agressions chirurgicales répétées pourraient entrainer la survenue d'une CE, en favorisant le passage d'allergène dans la paroi vésicale [4]. Sur le plan clinique, La symptomatologie de la CE est polymorphe et non spécifique. Les symptômes les plus fréquents étaient la pollakiurie (67%), la dysurie (62%), l'hématurie macroscopique ou microscopique (68%), la douleur sus-pubienne (49%) et la rétention aiguë d'urine (10%) [6, 9]. Chez les enfants, la CE peut se manifester par une énurésie secondaire [10]. Elle peut rarement se compliquer d'une perforation vésicale [11]. A un stade plus avancé, elle peut se manifester par certaines complications, notamment une urétéro-hydronéphrose secondaire à l'épaississement de la paroi vésicale, un reflux vésico-urétéral, des infections itératives ou encore une fistule entéro-vésicale [8]. L'examen clinique est généralement pauvre, il est normal dans la majorité des cas [12]. L'examen cytobactériologique des urines (ECBU) est peu contributif mais élimine une infection urinaire active [12]. La biologie montre une leucocytose avec éosinophilie dans 60 % des cas et parfois une éosinophilurie, même si celle-ci reste rare et non spécifique [13]. Une augmentation du taux sérique d'IgE et d'IgA est souvent retrouvée [8]. Le bilan parasitaire est le plus souvent négatif [12]. L'imagerie est peu contributive au diagnostic [8]. Elle peut mettre en évidence un syndrome tumoral sans préjuger de sa nature [14]. A la cystoscopie, la muqueuse vésicale est inflammatoire voire nécrosée et ulcérée. Elle est le siège de véritables formations polyploïdes faisant suspecter des tumeurs épithéliales en cas des formes pseudo-tumorales [15]. Le diagnostic formel est histologique, il s'agit d'un infiltrat inflammatoire dense riche en polynucléaires éosinophiles, qui diffuse dans un chorion fibro-'démateux et s'infiltre entre les faisceaux du muscle détrusor réalisant un tableau de pancystite de la muqueuse et de la sous muqueuse [4]. Les formes pseudo-tumorales sont marquées par le caractère discret de l'infiltrat superficiel contrastant avec la fréquence des lésions de nécrose et de fibrose des couches profondes [16]. Ce diagnostic ne peut être retenu qu'après avoir éliminé les autres conditions pouvant occasionner un état d'hyper-éosinophilie et en particulier les infections parasitaires (Schistosoma, toxocara etc…) [12]. D'où l'intérêt d'un examen parasitologique des urines et des selles ainsi qu'une cytologie urinaire [12]. En raison de la rareté de la CE, le traitement n'est pas standardisé et les modalités de traitement ainsi que le déroulement du traitement ont varié d'un cas à l'autre. Il consiste, en premier lieu, en la suppression de l'agent allergène s'il est retrouvé (médicament) associée à des anti-inflammatoires non stéroïdiens (AINS) [5]. Les corticoïdes sont utilisés deuxième intention, éventuellement associés à des antihistaminiques [4]. En cas de mauvaise réponse à la corticothérapie, un traitement par ciclosporine peut être proposé [17]. Cependant, les formes pseudo-tumorales semblent mal répondre à ces traitements [18]. De nombreux protocoles ont été proposés, mais sans succès probant: instillations de nitrate d'argent, d'azathioprine ou de mitomycine C [19]. En cas d'échec du traitement médical, ou en cas de complications, la chirurgie devient fondamentale [18]. Une résection endoscopique des lésions, une cystectomie partielle, voire une entérocystoplastie peuvent être discutées [18]. La surveillance est indispensable pour détecter une complication et évaluer l'efficacité du traitement [12]. Pour les formes pseudo-tumorales, P. Chaffange et coll. ont décrit une évolution inhabituelle, c'est la survenue d'une récidive suraiguë, moins d'un mois après une tumorectomie considérée comme complète [8]. La durée de cette surveillance n'est pas précisée mais il est légitime de préconiser une surveillance à long terme devant le risque de greffe d'une cystite interstitielle sur les lésions préexistantes [12, 16].

## Conclusion

La cystite éosinophile (CE) est une maladie rare avec infiltration trans-murale d'éosinophiles. Le diagnostic de la CE est histologique. Les patients atteints de CE présentent souvent des symptômes et des signes cliniques communs à d'autres troubles urologiques, tels qu'une infection des voies urinaires, une tumeur maligne et des symptômes urinaires plus faibles. Il n'existe pas de traitement normalisé, mais les traitements courants et efficaces comprennent les stéroïdes, les antihistaminiques et la chirurgie, souvent combinés.

### Etat des connaissances actuelles sur le sujet

- C'est une pathologie rare;
- La prise en charge thérapeutique n'est pas bien établie.

### Contribution de notre étude à la connaissance

- La surveillance régulière est nécessaire;
- La forme pseudo-tumorale est fréquente;
- Il n'existe pas de traitement normalisé, mais les traitements courants et efficaces comprennent les stéroïdes, les antihistaminiques et la chirurgie, souvent combinés.

## Conflits d’intérêts

Les auteurs ne déclarent aucun conflit d'intérêts.
